# Lamotrigine‐Associated Toxic Epidermal Necrolysis in a Southeast Asian Female: A Case Report Highlighting Sex and Ancestry‐Related Susceptibility

**DOI:** 10.1155/crps/2560198

**Published:** 2026-09-15

**Authors:** Sydney Christopherson, Naomi Graham, Jonathan Wirjo

**Affiliations:** ^1^ Department of Osteopathic Medicine, A.T. Still University Kirksville College of Osteopathic Medicine, Kirksville, Missouri, USA, atsu.edu; ^2^ Department of Psychiatry, Sunrise Graduate Medical Education Consortium, HCA Healthcare, Las Vegas, Nevada, USA, hcahealthcare.com

**Keywords:** case report, lamotrigine, stevens-johnson syndrome, toxic epidermal necrolysis

## Abstract

**Background:**

Stevens–Johnson syndrome (SJS) is a known, but rare adverse effect of Lamotrigine, occurring in less than 0.1% of the population. Rapid dose titrations of Lamotrigine, an antiepileptic used off‐label for the treatment of bipolar disorder, have been shown to increase the risk of SJS, and in even rarer cases, progression to toxic epidermal necrolysis (TEN), which currently has too few cases to report an estimated incidence. Data suggest that additional factors, including female sex and certain HLA alleles associated with East and Southeast Asian ancestries, may be associated with increased risk of Lamotrigine‐induced SJS/TEN.

**Case presentation:**

This case presents a 26‐year‐old Southeast Asian female with bipolar I disorder who developed SJS shortly after her Lamotrigine dose was titrated appropriately from 50 milligrams (mg) to 100 mg daily, which rapidly progressed to TEN. The patient received treatment, including steroids, intravenous (IV) immunoglobulin, and surgical debridement, and was discharged home in stable condition after 8 days.

**Conclusion:**

This case prompts consideration of sex and ancestry when identifying high‐risk populations taking Lamotrigine. Continued research into sex‐ and ancestry‐based pharmacogenomics may reduce the incidence of these life‐threatening reactions.

## 1. Introduction

Stevens–Johnson syndrome (SJS) and toxic epidermal necrolysis (TEN) represent a life‐threatening spectrum of desquamating rashes of the skin and mucous membranes [1]. These rare dermatologic emergencies are associated with significant morbidity and mortality. Estimated mortality rates from 2010 to 2020 were 5.4% and 15.3% for SJS and TEN, respectively [2]. These conditions are commonly associated with medication use, including Lamotrigine, an antiseizure medication, which is widely used off‐label as a mood stabilizer to treat bipolar disorder [3].

Lamotrigine‐associated SJS and TEN most often occur 2–8 weeks after medication initiation. In adults, studies report up to a 0.08% incidence of Lamotrigine‐associated SJS, and as of 2023, the number of cases of Lamotrigine‐associated TEN was too low to estimate an incidence [4]. Further, randomized control trials evaluating adverse skin reactions in patients started on Lamotrigine specifically for the treatment of bipolar disorder showed an incidence of 0.02% [4]. Although uncommon, these reactions carry significant clinical consequences. Certain factors have been shown to increase the risk of Lamotrigine‐associated SJS/TEN. Severe cutaneous reactions are more likely when Lamotrigine is initiated at an elevated dose, rapidly titrated, or co‐administered with valproic acid [5]. Additionally, a 2018 meta‐analysis supported an ancestry‐specific genetic risk of Lamotrigine‐induced SJS/TEN, concentrated in certain East/Southeast Asian populations [6]. Lastly, in previously reported cases, females were more frequently affected than males [7].

Despite their rarity, SJS and TEN can be life‐threatening, making recognition of risk factors essential for clinical decision‐making and patient education.

## 2. Case Presentation

A 26‐year‐old Southeast Asian female with a history of bipolar I disorder presented with a chief complaint of a pruritic skin rash. On presentation to the emergency department, she reported the onset of a rash the day prior, a few hours after titrating Lamotrigine from 50 milligrams (mg) to 100 mg daily. At rash onset, the patient took over‐the‐counter antihistamines and was seen at urgent care, given an oral steroid pack. The next day, the rash became increasingly uncomfortable, pruritic, and with mucocutaneous involvement, prompting her to present to the emergency department.

Upon evaluation of pertinent history, the patient explained she was diagnosed with bipolar I disorder 7 months prior. She was previously taking lithium as a mood stabilizer but switched to Lamotrigine due to reported weight gain. Per patient report, her outpatient provider appeared to have followed standard titration guidelines, starting at 25 mg daily for 2 weeks, increasing to 50 mg daily for an additional 2 weeks, and then to 100 mg daily shortly before the onset of her rash. No concomitant medications known to alter Lamotrigine metabolism, including valproic acid, were identified. Information regarding oral contraceptive use was unavailable.

On admission, physical examination revealed a widespread erythematous maculopapular and vesicular eruption involving ~80%–85% of her body surface area, including the face, trunk, palms, soft palate, buccal mucosa, and bilateral upper and lower extremities, as seen in Figures 1–5.

**Figure 1:**
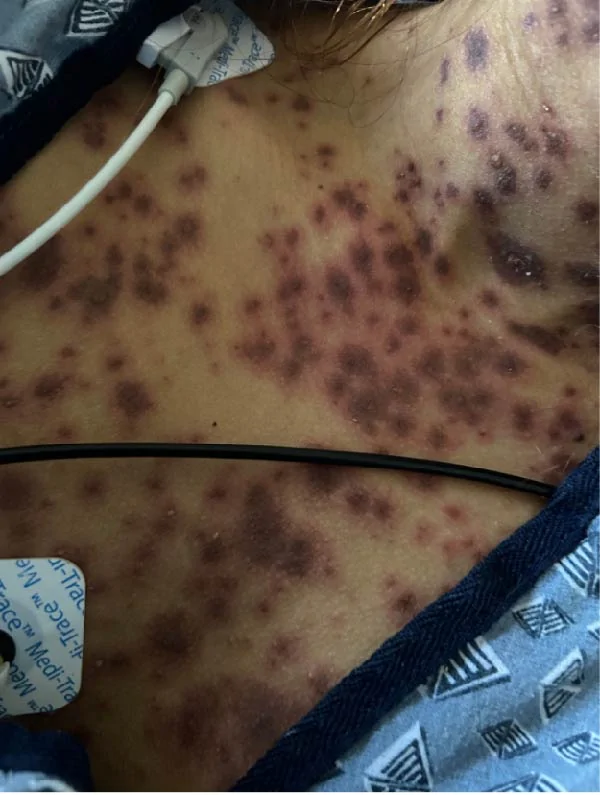
Patient’s dermatologic findings on her chest and neck on day 2 of hospitalization.

**Figure 2:**
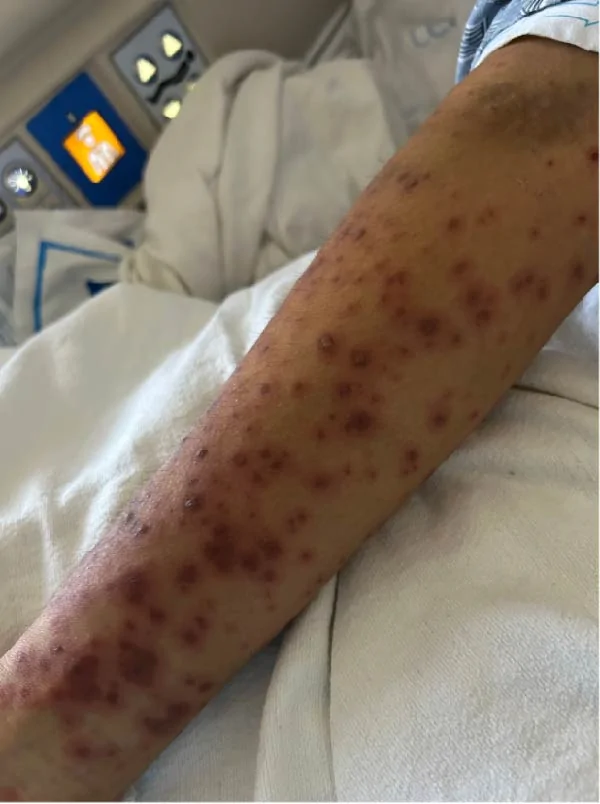
Patient’s dermatologic findings on her left upper extremity day 2 of hospitalization.

**Figure 3:**
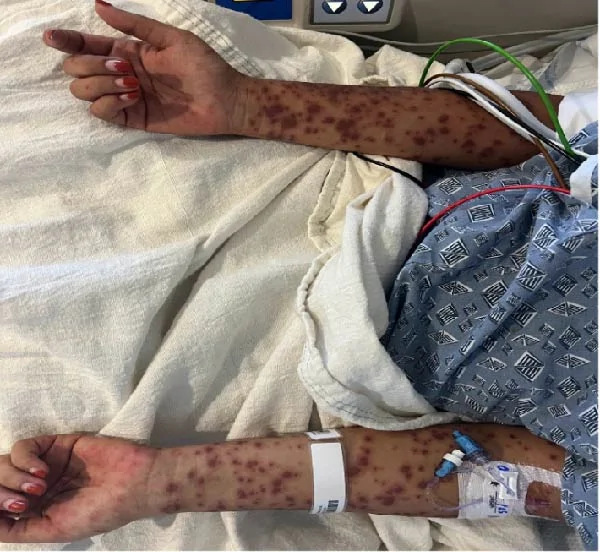
Patient’s dermatological findings on her bilateral upper extremities on day 6 of hospitalization.

**Figure 4:**
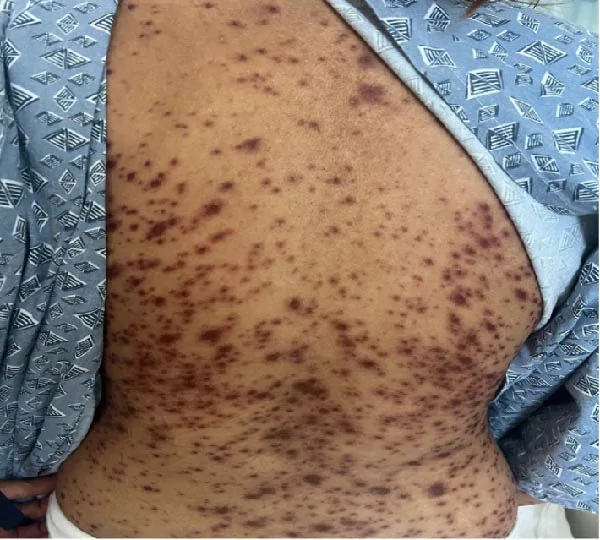
Patient’s dermatological findings on her back on day 6 of hospitalization.

**Figure 5:**
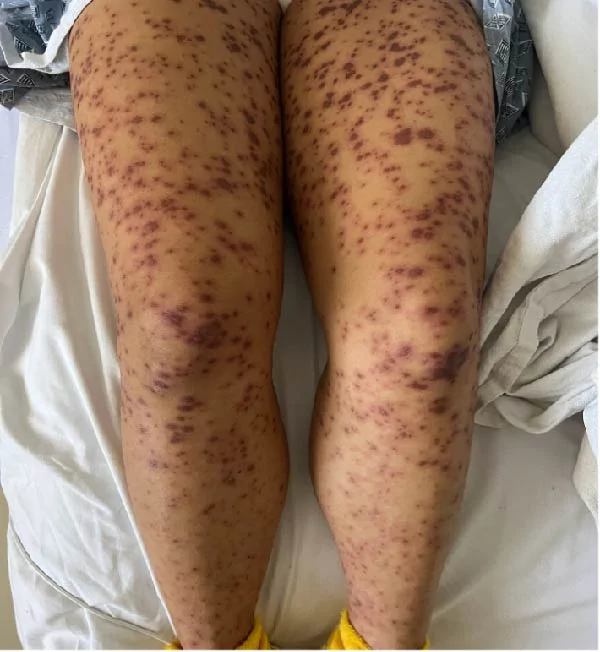
Patient’s dermatological findings on her bilateral lower extremities on day 6 of hospitalization.

Lamotrigine was immediately discontinued, and the patient was admitted to the burn intensive care unit (ICU) for presumptive diagnosis of SJS/TEN. Her treatment included intravenous (IV) methylprednisone 40 mg twice a day, IV immunoglobulin (IVIG) 40 g daily, topical and IV antibiotics, and surgical debridement of necrotic lesions on her face, neck, and chest. On hospital day 5, skin punch biopsy results confirmed the diagnosis of TEN. After 8 days in the ICU, the patient was discharged home in stable condition on prednisone 40 mg daily.

Long‐term follow‐up information was not available for this report. The patient was discharged with outpatient follow‐up through burn surgery, dermatology, and psychiatry services. Therefore, the presence or absence of sequelae, including ocular, skin, or psychiatric sequelae, could not be determined and is a limitation of this report.

HLA genotyping was not performed during hospitalization, as the focus was on acute patient stabilization, and the FDA does not specifically recommend retrospective HLA testing after a lamotrigine‐associated adverse reaction. Because HLA testing is also not required before initiating lamotrigine, its absence should not be interpreted as a deviation from standard care, and genotyping should not replace appropriate clinical monitoring and treatment of cutaneous adverse effects. However, the lack of testing limits our ability to determine whether pharmacogenomic factors contributed to her clinical presentation.

## 3. Discussion

The current recommended maintenance dose for Lamotrigine in the treatment of bipolar disorder is 200 mg daily. According to the Food [8] and Drug Administration (FDA), slow titration is suggested to reduce the risk of severe cutaneous reactions, starting with 25 mg daily for 2 weeks, followed by 50 mg daily for 2 weeks, then 100 mg daily during the fifth week, and finally reaching the target dose of 200 mg daily by week six. Since the introduction of this titration schedule in 1994, the incidence of severe cutaneous reactions has declined from ~1% to 0.1%–0.01% [9]. Despite adherence to the standard titration schedule, this patient unfortunately developed TEN. We believe this warrants attention, as it may suggest that additional risk factors, such as sex and ethnicity, might influence a patient’s risk.

### 3.1. Female Predominance

Several case series and analyses have reported a female predominance among Lamotrigine‐associated SJS/TEN cases. A 2024 retrospective study showed a female‐to‐male ratio of 2.7:1 [10], while another 2024 analysis found that 92% of affected patients were female [11]. Although randomized controlled trials are lacking, the consistent observation of female predominance shows that women represent a higher‐risk population when taking this medication.

There are multiple factors that may contribute to the observed female predominance. First, Lamotrigine is primarily metabolized via UDP‐glucuronosyltransferase (UGT)‐mediated glucuronidation, predominately the UGT1A4 and UGT2B10 isoforms. Sex‐specific differences in UGT enzyme activity have been demonstrated, with polymorphisms in UGT2B15, UGT2B17, UGT1A4, and UGT2B7 exerting a larger impact on Lamotrigine metabolism in women than in men [12–14].

Additionally, endogenous estrogen and progesterone fluctuations during the menstrual cycle can influence Lamotrigine metabolism via induction of hepatic glucuronidation. Exogenous hormones, such as oral contraceptives, have been shown to reduce plasma levels of Lamotrigine by 50% via UGT induction. Therefore, during certain phases of the menstrual cycle or in the absence of exogenous hormones, lower drug clearance may lead to higher plasma concentrations [15]. Further, because women are prescribed Lamotrigine more frequently than men, exposure bias could contribute to the observed sex disparity [14]. Collectively, pharmacokinetic differences, hormonal influences, and prescribing patterns likely contribute to the observed female predominance.

### 3.2. Susceptibility Linked to Ancestry

In addition to sex‐specific factors, genetic susceptibility related to ancestry may contribute to the risk of Lamotrigine‐associated SJS/TEN. Human leukocyte antigen (HLA) alleles *B* ^∗^15:02, *B* ^∗^31:01, and *B* ^∗^44:03, involved in antigen presentation and T‐cell activation, have demonstrated associations with antiepileptic drug‐induced SJS/TEN [6, 8]. A 2024 review by Tham et al. confirmed an association between HLA‐*B* ^∗^15:02 and Lamotrigine‐associated SJS/TEN, and additional evidence has identified HLA‐*A* ^∗^24:02 as an independent and additive risk factor, with increased risk observed among individuals carrying both HLA‐*A* ^∗^24:02 and HLA‐*B* ^∗^15:02 [16, 17]. A 2022 meta‐analysis further supported the role of HLA variation in Lamotrigine‐associated SJS/TEN, demonstrating increased risk among carriers of susceptibility alleles in both the HLA‐A and HLA‐B loci with an odds ratio greater than 2 [18].

The HLA alleles linked to Lamotrigine‐associated SJS/TEN are particularly common among individuals of East and Southeast Asian ancestry. HLA‐*B* ^∗^15:02 occurs in ~6%–8% of Southeast Asian populations but is rare among European ancestry [6, 17]. Country‐specific allele frequencies show that HLA‐*B* ^∗^15:02 allele prevalence may exceed 15% in populations throughout Vietnam, Thailand, and Malaysia [19, 20]. Additionally, the HLA‐*A* ^∗^24:02 allele is highly prevalent in similar populations, compounding susceptibility in these patients [19, 21].

The FDA‐approved prescribing information recognizes an association between HLA‐B15:02 and an increased risk of lamotrigine‐associated SJS/TEN in certain populations of Asian ancestry, including Han Chinese and Thai populations. However, pretreatment HLA‐B15:02 genotyping is not required before initiating lamotrigine. Instead, the FDA recommends weighing the potential risks and benefits of therapy in patients known to carry HLA‐*B* ^∗^15:02 and emphasizes that genotyping should not replace appropriate clinical vigilance and patient management [8, 21].

Because of this, routine pretreatment screening has been initiated in countries such as Taiwan and Singapore, and cost‐effectiveness has been demonstrated across multiple Southeast Asian countries, making genotype analysis a favorable strategy in reducing risk for these populations [22, 23].

Although HLA alleles contribute to risk, genotyping should not be considered a definitive tool for predicting Lamotrigine‐associated SJS/TEN. Many HLA‐*B* ^∗^15:02 positive patients can tolerate Lamotrigine without developing cutaneous reactions, and SJS/TEN can occur in patients who do not carry known risk alleles. Therefore, HLA testing should complement, but not replace, patient counseling, recognition of risk factors, and clinical monitoring during dose titration.

### 3.3. Recommendations for Risk‐Adjusting Monitoring

Despite the growing evidence supporting both HLA and sex‐related risks, there are no formal guidelines recommending individualized Lamotrigine titration beyond HLA testing, so this case highlights potential areas for future investigation [8]. Therefore, these proposed approaches should be considered clinical considerations rather than evidence‐based guideline recommendations.

For patients with increased susceptibility, future studies could evaluate whether enhanced monitoring strategies improve early recognition of cutaneous adverse reactions. Potential approaches include more frequent follow‐up during the first 2–8 weeks of therapy, when the risk of SJS/TEN is greatest, early telehealth or other remote check‐ins following treatment initiation and dose escalation to identify evolving rash or mucosal symptoms, and reassessment at each dose increase. Patient education should emphasize prompt reporting of rash, mucosal lesions, fever, facial swelling, or other symptoms concerning for cutaneous adverse reactions.

In addition, prospective research is needed to determine if lower starting doses or slower titration schedules could reduce risk in populations with suspected increased susceptibility, such as with high‐risk HLA alleles. This type of approach would require careful evaluation because slow titration may delay therapeutic effects, and the optimal risk‐stratified dosing is unknown. Until prospective studies establish whether these strategies improve outcomes, standard FDA‐recommended dosing, careful patient counseling, and prompt evaluation of symptoms remain the evidence‐supported approach.

### 3.4. Report Strengths and Weaknesses

Strengths of this case report include clear documentation of appropriate drug titration, symptom onset, and dermatologic findings. Limitations include the inability of a single case to establish causality or generalizability, the absence of confirmatory genetic screening for high‐risk HLA alleles, and limited long‐term outcome data due to the inpatient setting.

## 4. Conclusion

This case highlights the contribution of sex‐ and ancestry‐related factors that may predispose patients to Lamotrigine‐associated SJS/TEN. Although slow dose escalation has reduced the incidence of severe cutaneous reactions, it does not completely eliminate risk, particularly among patients with pharmacokinetic and pharmacogenomic susceptibility. Therefore, demographic risk factors, particularly female sex and East and Southeast Asian ancestry, should be considered by clinicians when initiating Lamotrigine. Future studies are needed to evaluate risk‐stratified approaches to Lamotrigine prescribing, but in the meantime, clinical vigilance, patient education, and early evaluation of cutaneous symptoms remain critical strategies to reduce morbidity and mortality associated with SJS/TEN.

**Nomenclature**
SJS: Stevens‐Johnson syndrome
TEN: toxic epidermal necrolysis
Mg: milligrams
ICU: intensive care unit
IV: intravenous
IVIG: intravenous immunoglobulin
FDA: Food and Drug Administration
HLA: human leukocyte antigen
CTL: cytotoxic T lymphocytes
NK: natural killer
UGT: UDP‐glucuronosyltransferase.

## Author Contributions

Sydney Christopherson was the primary author. Naomi Graham and Jonathan Wirjo oversaw patient care, edited the manuscript, and approved the final draft.

## Funding

No funding received.

## Disclosure

All scientific content, interpretation, and final editing were reviewed and approved by the authors.

## Ethics Statement

Institutional Review Board approval was not required for this single case report in accordance with institutional policy.

## Consent

Written informed consent was obtained from the patient for publication of this case report and any accompanying images. A copy of the written consent is available for review by the Editor‐in‐Chief of this journal.

## Conflicts of Interest

The authors declare no conflicts of interest.

## Data Availability

Data sharing are not applicable to this article as no datasets were generated or analyzed during the current study.
